# Metformin modulates survival, oxidative stress, and gene expression in a high-sucrose *Drosophila* diabetes model

**DOI:** 10.1371/journal.pone.0359638

**Published:** 2026-10-01

**Authors:** Mohamed Lotfy, Fatema Al Sawai, Dhoha Alyammahi, Biduth Kundu

**Affiliations:** Biology Department, College of Science, United Arab Emirates University, Al Ain, United Arab Emirates; BSRC Alexander Fleming: Biomedical Sciences Research Center Alexander Fleming, GREECE

## Abstract

**Background:**

Excess dietary sugar is a major contributor to metabolic disorders, including type 2 diabetes mellitus (T2DM). This study investigates the physiological, biochemical, behavioral, and molecular effects of high-sucrose diets in *Drosophila melanogaster* and evaluates the modulatory effects of metformin under these conditions.

**Methods:**

Flies were reared on normal and high-sucrose diets (5% and 15%), with or without metformin treatment (5 mM and 15 mM). Pupation, survival, locomotor activity (negative geotaxis), trehalose levels, antioxidant enzyme activities (SOD, GST), and expression of stress-related genes (Mn-SOD, Cu/Zn-SOD, MTH, CAT, Hsp40, Hsp70) were assessed after 10 days of treatment. Gene expression levels were quantified relative to the normal control group using RpS20 as the housekeeping reference.

**Results:**

High-sucrose diets significantly reduced pupation and survival rates, impaired locomotor performance, increased trehalose levels, and decreased antioxidant enzyme activities. These diets were also associated with downregulation of stress- and antioxidant-related genes. Metformin treatment partially attenuated several of these alterations relative to the corresponding sucrose-treated groups, including improvements in pupation, survival, locomotor performance, metabolic parameters, antioxidant enzyme activity, and gene expression.

**Conclusion:**

High-sucrose diets induced metabolic disturbances in *Drosophila melanogaster*, including impaired developmental progression and survival, functional locomotor impairment, carbohydrate imbalance, oxidative stress, and suppression of stress-related gene expression. Metformin partially attenuated several of these changes within the corresponding sucrose-treatment groups. These findings support the utility of *Drosophila melanogaster* as a tractable model for studying diet-induced metabolic dysfunction and evaluating pharmacological interventions.

## Introduction

Diabetes mellitus (DM) is a chronic metabolic disorder marked by persistent hyperglycemia due to insufficient insulin secretion, impaired insulin action, or both. Under normal conditions, insulin-a hormone produced by the pancreas-facilitates the uptake of glucose from the bloodstream into cells for energy. In diabetes, this process is disrupted, leading to elevated blood glucose levels [1].

If left uncontrolled, chronic hyperglycemia can lead to severe complications affecting the cardiovascular system, kidneys, retina, and nervous system, significantly impacting quality of life and increasing the risk of premature mortality. The global prevalence of diabetes is rapidly increasing, with more than 537 million adults affected in 2021 and projections exceeding 592 million by 2035 [2]. While genetic predisposition contributes to disease risk, lifestyle factors such as physical inactivity and diets high in fat and sugar play a significant role in its onset and progression [3].

Type 2 diabetes is the most common form and is closely associated with obesity and insulin resistance. Excessive intake of dietary sugars has been linked to metabolic dysfunction, making dietary habits a major modifiable risk factor [4]. Alarmingly, type 2 diabetes is increasingly diagnosed in younger individuals, often progressing silently in its early stages [5].

Complications of diabetes include macrovascular issues like cardiovascular disease and stroke, as well as microvascular complications such as nephropathy, retinopathy, and neuropathy [6]. A central mechanism in the development of these complications is oxidative stress induced by prolonged hyperglycemia. This stress arises from pathways like glucose auto-oxidation, protein glycation, and the formation of advanced glycation end products (AGEs), which collectively generate reactive oxygen species (ROS) that overwhelm cellular antioxidant defences and cause tissue damage [7].

In recent years, *Drosophila melanogaster* has gained prominence as a model organism for studying metabolic disorders. The fruit fly possesses conserved insulin signalling pathways, a short life cycle, and is highly amenable to genetic manipulation, making it an effective system for exploring the molecular basis of diabetes and testing potential therapeutic interventions [8].

*D. melanogaster* has emerged as a powerful model for studying diabetes mellitus, owing to its highly conserved insulin/insulin-like growth factor (insulin/IGF) signalling pathway, genetic tractability, and metabolic parallels with humans [9,10]. The fly model replicates many core features of type 2 diabetes, including hyperglycemia, insulin resistance, and lipid accumulation, particularly when exposed to high-sugar diets [11,12].

High-sucrose feeding in *Drosophila* elevates circulating glucose and insulin-like peptides (ILPs), initially enhancing insulin signalling. However, prolonged exposure induces insulin resistance-mirroring early stages of type 2 diabetes in humans [13]. This resistance is especially evident in the fat body, the fly’s functional equivalent of the liver and adipose tissue, where impaired insulin sensitivity disrupts glucose uptake and storage [14].

High-sucrose feeding in *Drosophila* has been consistently shown to disrupt systemic carbohydrate homeostasis through dysregulation of *Drosophila* insulin-like peptides (DILPs), particularly DILP2, DILP3, and DILP5. In flies, insulin signaling is mediated by DILPs that activate the insulin receptor (InR), triggering the downstream PI3K-AKT cascade and modulating effectors such as dFOXO and d4EBP. This signaling axis is functionally analogous to mammalian insulin and insulin-like growth factor (IGF) pathways. Genetic ablation of insulin-producing cells or disruption of InR signaling results in elevated circulating sugars, including trehalose, confirming the tight regulatory relationship between DILP signaling and hemolymph carbohydrate levels [15,16]. Chronic high-sucrose exposure impairs insulin pathway responsiveness, leading to reduced AKT activation, altered dFOXO activity, and sustained trehalose accumulation-hallmarks of insulin resistance in this model [13,17]. Thus, the conserved DILP-InR-AKT-dFOXO axis provides a mechanistic framework through which diet-induced metabolic dysfunction and insulin/IGF-like signaling disruption can be investigated in *Drosophila* and supports the use of trehalose quantification as a validated functional surrogate of insulin pathway impairment [11]

In addition to metabolic dysfunction, high-sugar diets in flies result in increased body fat, delayed development, reduced fertility, and shortened lifespan-further aligning the model with human diabetic pathophysiology [11,18]. These outcomes make sucrose-rich diets a reliable and reproducible method for inducing diabetic-like phenotypes in both longitudinal and interventional studies.

Compared to vertebrate models, *Drosophila* offers several advantages, including a short generation time, low maintenance costs, and advanced genetic tools such as the GAL4/UAS and RNAi systems. These enable precise manipulation of gene expression to study gene-diet interactions, oxidative stress, mitochondrial function, and the regulation of metabolic genes during diabetes progression [19]. The simplified insulin signalling cascade in flies also reduces redundancy, facilitating clearer mechanistic insights [20].

Despite its simplicity, *Drosophila* complements mammalian models in diabetes research. Flies fed high-sugar diets reliably exhibit hallmarks of human metabolic disorders, making them a valuable system for screening antidiabetic compounds and investigating the molecular basis of diet-induced insulin resistance and metabolic stress. Furthermore, the model has proven effective for exploring how dietary factors influence redox balance, stress responses, and metabolic gene expression [11,12].

Metformin, a biguanide compound, is a widely prescribed first-line treatment for type 2 diabetes mellitus (T2DM). Its primary mechanism involves lowering hepatic glucose production and enhancing insulin sensitivity in peripheral tissues [21]. At the cellular level, metformin inhibits mitochondrial complex I, leading to an increased AMP/ATP ratio that activates AMP-activated protein kinase (AMPK)-a central energy sensor that regulates glucose and lipid metabolism [22,23].

Activation of AMPK promotes glucose uptake, particularly through GLUT4 translocation, and suppresses the mechanistic target of rapamycin (mTOR) pathway, which is involved in protein synthesis, cell growth, and autophagy [24,25]. Through these actions, metformin improves insulin signalling, enhances energy homeostasis, and helps cells adapt to metabolic stress. Furthermore, under conditions of impaired insulin signalling, AMPK activation upregulates stress-responsive genes, contributing to improved cellular resilience [26,27].

In addition to its metabolic effects, metformin exhibits significant antioxidant properties. It enhances the activity and expression of key antioxidant enzymes such as superoxide dismutase (SOD1), catalase (CAT), and peroxiredoxin 6 (Prdx6), while reducing intracellular levels of reactive oxygen species (ROS) [28,29]. These effects help counter oxidative stress-a major contributor to diabetic complications.

In *D. melanogaster*, metformin has been shown to alleviate oxidative stress and improve stress resistance, particularly in models with compromised antioxidant defences, such as SOD1-deficient mutants [30]. These findings support the utility of *Drosophila* in exploring the dual metabolic and redox-modulatory effects of metformin, and in evaluating its potential for therapeutic interventions targeting diabetes-related oxidative damage.

This study investigates the modulatory effects of metformin in a *D. melanogaster* model of diet-induced metabolic dysfunction caused by high-sucrose feeding. By examining survival and pupation rates, locomotor performance, trehalose levels, antioxidant enzyme activities (superoxide dismutase [SOD] and glutathione S-transferase [GST]), and the expression of key stress-related genes (manganese superoxide dismutase [Mn-SOD], copper/zinc superoxide dismutase [Cu/Zn-SOD], methuselah [MTH], catalase [CAT], heat shock protein 40 [Hsp40], and heat shock protein 70 [Hsp70]), the study evaluates whether metformin attenuates metabolic and oxidative stress under each predefined sucrose condition. The findings provide an integrated assessment of physiological, biochemical, behavioral, and molecular responses. The findings will provide insight into the mechanisms by which metformin improves metabolic health and stress resilience, highlighting the utility of *Drosophila* as an effective model for studying antidiabetic interventions.

## Materials and methods

### *D*. *melanogaster* strain and rearing

Wild-type *Drosophila melanogaster* (Oregon R strain) was originally obtained from Carolina Biological Supply (Burlington, NC, USA) and is maintained in the Genetics Laboratory, Biology Department, United Arab Emirates University. Flies were reared on instant *Drosophila* medium (Formula 4–24, Carolina Biological Supply) supplemented with dry yeast under controlled environmental conditions at 23 ± 1°C with a 12:12 h light/dark cycle. Adult flies were transferred to fresh food vials every four days to maintain optimal nutrition and prevent overcrowding. These rearing conditions were maintained throughout all experiments.

### Experimental design and grouping

To ensure sufficient biological material for all endpoints and to avoid confounding effects related to mortality, behavioral testing, and destructive downstream assays, independent parallel cohorts were established for survival assessment, pupation analysis, locomotor performance, biochemical assays, and gene expression analysis. Thus, each endpoint was evaluated using a dedicated experimental cohort rather than relying on the same surviving flies for all subsequent measurements.

Flies were randomly assigned to five experimental groups:

(1) Normal control (NC): standard diet containing 0% sucrose
(2) 5% sucrose (S5): diet containing 5% sucrose
(3) 15% sucrose (S15): diet containing 15% sucrose
(4) 5% sucrose + 5 mM metformin (SM5): diet containing 5% sucrose supplemented with 5 mM metformin
(5) 15% sucrose + 15 mM metformin (SM15): diet containing 15% sucrose supplemented with 15 mM metformin

For each adult endpoint, each experimental condition consisted of three independent biological replicate vials, with 20 flies per vial (10 males and 10 virgin females). All treatment groups were established simultaneously within each experimental run and were maintained under identical environmental conditions. The complete experiment was independently repeated on three separate occasions using newly established fly cohorts. All treatments were administered for 10 days. Sucrose diets were prepared from analytical-grade sucrose dissolved in distilled water and diluted to the required concentrations before incorporation into the food medium. Metformin hydrochloride (Sigma-Aldrich, USA) was prepared as a 100 mM stock solution and added to the food to achieve final concentrations of 5 mM or 15 mM as indicated. Food vials were freshly prepared and replaced every 48 hours during the treatment period. Experimental conditions and dietary supplementation were adapted from previously established methods in *D. melanogaster* [31–34] by incorporating the two predefined sucrose conditions and their corresponding metformin treatments and by using independent cohorts for each endpoint to avoid confounding from mortality, repeated behavioral testing, or destructive sampling.

### Pupation rate assessment

Pupation rate was assessed using a separate synchronized developmental cohort. For each treatment group, adult flies were transferred to freshly prepared food media and allowed to oviposit during the same defined collection period to generate synchronized embryos. An equal number of embryos was placed in each of three independent biological replicate vials per group. Vials were maintained under standard laboratory conditions (23 ± 1°C, 12:12 h light/dark cycle, 60% humidity). The larvae remained in the same developmental vials throughout the assay and were not transferred during development or subjected to the 48-hour adult food-replacement schedule. At the end of the observation period, the total number of visible pupae in each vial was recorded, and pupation rate was calculated as: pupation rate (%) = (number of pupae/initial number of embryos) × 100. The mean was calculated from the three independent replicate vials. The procedure was adapted from Sakaguchi and Suzuki [35] by incorporating the experimental sucrose and metformin conditions directly into the developmental medium and by using synchronized embryo collections to minimize developmental variation among groups. The control group represents the observed biological baseline rather than a theoretical 100% developmental success rate.

### Survival rate assessment

A dedicated adult cohort was used for survival analysis. Flies were maintained on their assigned experimental diets for 10 days under controlled conditions. Mortality was recorded every 48 hours at the time of food replacement. Flies were considered dead when they failed to respond to gentle stimulation with a fine paintbrush. Food was replaced every 48 hours, but flies remained within their assigned replicate cohorts and were not redistributed among treatment vials. On day 10, survival rate was calculated as the number of live flies divided by the initial number of flies per vial, multiplied by 100 [36,37].

### Negative geotaxis and locomotor performance

Locomotor performance was evaluated using the negative geotaxis assay in a separate adult cohort on Day 10. Each assay used a mixed-sex group of 20 flies (10 males and 10 virgin females), preserving the same sex ratio across all treatment groups. Flies were placed in vertical glass tubes and allowed to acclimate for 30 min. All assays were conducted during the light phase under uniform ambient laboratory illumination, with identical lighting conditions maintained across treatment groups and biological replicates. They were then gently tapped to the bottom, and climbing success was quantified as the percentage of flies crossing the 8-cm mark within 10 s. Each biological replicate was tested three times, with sufficient recovery time between trials, and the mean of the three trials was used for analysis. Climbing behavior was recorded for 20 s, and video playback was paused at the 10-s time point. The assay was adapted from previous studies [36,38] by applying a standardized 8-cm threshold and 10-s endpoint to mixed-sex cohorts after chronic dietary treatment, thereby allowing consistent comparison of locomotor performance among groups.

### Biochemical assays

#### Sample preparation.

Biochemical markers were assessed in a dedicated adult cohort after 10 days of dietary treatment. For each group, flies were anesthetized using FlyNap and homogenized in 100 µL of cold phosphate-buffered saline (PBS, pH 7.4) using a motorized pestle. Homogenates were centrifuged at 1000 × g for 5 min at 4°C, and the supernatants were collected and maintained on ice until analysis.

#### Trehalose quantification.

Trehalose, the primary circulating sugar in *D. melanogaster*, was quantified as an indicator of carbohydrate metabolism and hyperglycemia-like metabolic disturbance [13,39]. After centrifugation, each whole-fly homogenate was divided into two aliquots. In the first aliquot, free glucose was measured directly using a glucose oxidase-based colorimetric assay (Pointe Scientific, USA), and absorbance was read at 500 nm according to the manufacturer’s protocol. The second aliquot was incubated with trehalase to hydrolyze trehalose into glucose, after which total glucose concentration (free glucose plus trehalose-derived glucose) was measured using the same assay. Trehalose-derived glucose was calculated by subtracting the initial free-glucose value from total glucose measured after trehalase digestion, and final values were determined using a glucose standard curve. The method was adapted from Nunes and Drummond-Barbosa [40] by using whole-adult-fly homogenates and paired aliquots from the same biological sample, which minimized sample-to-sample variation and permitted calculation of trehalose-derived glucose under the dietary conditions tested.

#### Superoxide dismutase (SOD) activity.

SOD activity was measured as an index of antioxidant defense using a commercial SOD Assay Kit (Sigma-Aldrich, USA), based on inhibition of xanthine oxidase-mediated color formation. Absorbance was measured at 440 nm. Total protein concentration was determined using the Bradford Protein Assay Kit (Bio-Rad, USA), with absorbance recorded at 595 nm. SOD activity was normalized to total protein content and expressed as units per milligram of protein (U/mg protein). The procedure was adapted from published antioxidant-assay methods [41,42] by applying the commercial assay to whole-adult-fly homogenates and normalizing activity to Bradford-measured protein concentration, allowing quantitative comparison among dietary treatment groups.

#### Glutathione-S-transferase (GST) activity.

GST activity was determined by measuring the enzyme-catalyzed conjugation of 1-chloro-2,4-dinitrobenzene (CDNB) with reduced glutathione (GSH), which forms a detectable thioether product. The increase in absorbance at 340 nm was monitored to calculate enzyme activity using a molar extinction coefficient of 9.6 mM^-1·cm^-1 for CDNB. GST activity was normalized to protein concentration and expressed as milliunits per milligram of protein (mU/mg protein). The protocol was adapted from Ecker et al. [33] by using whole-adult-fly homogenates generated under the present dietary treatments and by normalizing GST activity to total protein concentration to facilitate comparison among experimental groups.

#### Quantitative real-time PCR gene expression analysis.

Gene expression analysis was performed in a separate dedicated cohort following 10 days of treatment. For each biological replicate, 10 males and 10 females were randomly collected from the endpoint-specific cohort and pooled for RNA extraction. Flies were preserved in RNAlater and stored at −80°C until processing. Total RNA was extracted from whole flies using the RNeasy Protect Kit (Qiagen, TX, USA). Complementary DNA (cDNA) was synthesized using the High-Capacity cDNA Reverse Transcription Kit (Thermo Fisher Scientific, MA, USA). Quantitative real-time PCR (qRT-PCR) was conducted using an Applied Biosystems QuantStudio 5 Real-Time PCR System and PowerUp SYBR Green Master Mix (Thermo Fisher Scientific) with gene-specific primers listed in Table 1. Relative gene expression was calculated using the 2^-ΔΔCt method. RpS20 was used as the housekeeping reference gene for normalization, and the normal control (NC) group served as the calibrator and was assigned a relative expression value of 1. Expression levels in S5, S15, SM5, and SM15 were therefore expressed relative to the NC group [37]. Gene expression was assessed at a single endpoint rather than longitudinally. Target genes included antioxidant defense genes (Mn-SOD, Cu-Zn-SOD, and CAT) and stress/proteostasis-related genes (MTH, Hsp40, and Hsp70).

**Table 1:** Primer sequences used for qRT-PCR.

Genes	Forward Primers (5’ to 3’)	Reverse Primers (5’ to 3’)
**RpS20**	CCGCATCACCCTGACATCC	TGGTGATGCGAAGGGTCTTG
**Mn-SOD**	CACATCAACCACACCATCTTC	CGTCTTCCACTGCGACTC
**Cu-Zn-SOD**	GCGGCGTTATTGGCATTG	ACTAACAGACCACAGGCTATG
**MTH**	AGCGTATATTAGGAGTGAAGAAGG	CCGTAGGAAGAAGGTGTAAGTC
**CAT**	TGAACTTCCTGGATGAGATGTC	TCTTGGCGGCACAATACTG
**Hsp40**	GAGATCATCAAGCCCACCACAAC	CGGGAAACTTAATGTCGAAGGAGAC
**Hsp70**	TCGATGGTACTGACCAAGATGAAGG	GAGTCGTTGAAGTAGGCTGGAACTG

### Statistical analysis

All data are presented as mean ± standard error of the mean (SEM). Statistical analyses were performed using GraphPad Prism version 9.0 (GraphPad Software, USA). Comparisons among experimental groups were performed using one-way analysis of variance (ANOVA) followed by Tukey’s multiple-comparison post hoc test. A value of P < 0.05 was considered statistically significant. Because the experimental design consisted of matched sucrose-metformin intervention groups rather than a full factorial sucrose × metformin design, statistical interpretation was restricted to NC versus S5, NC versus S15, S5 versus SM5, and S15 versus SM15. No statistical inference was made regarding the independent effect of metformin dose or an interaction between sucrose concentration and metformin concentration. In the figures, * indicates a significant difference relative to NC, and # indicates a significant difference between a sucrose-only group and its corresponding metformin-treated group.

## Results

### Pupation rate of *D. melanogaster*

The normal control (NC) group exhibited the highest pupation rate, as shown in Fig 1. Exposure to sucrose-containing diets significantly impaired developmental progression, as evidenced by reduced pupation in both sucrose-fed groups. Flies fed 5% sucrose (S5) showed a moderate but significant decline in pupation rate, whereas those fed 15% sucrose (S15) exhibited a more pronounced significant reduction. Metformin treatment partially attenuated these effects. The SM5 group displayed a slight but non-significant increase in pupation relative to S5, whereas the SM15 group showed a significant improvement relative to S15. These findings indicate that high-sucrose diets impair developmental success and that metformin partially improves pupation within the corresponding sucrose condition.

**Fig 1:**
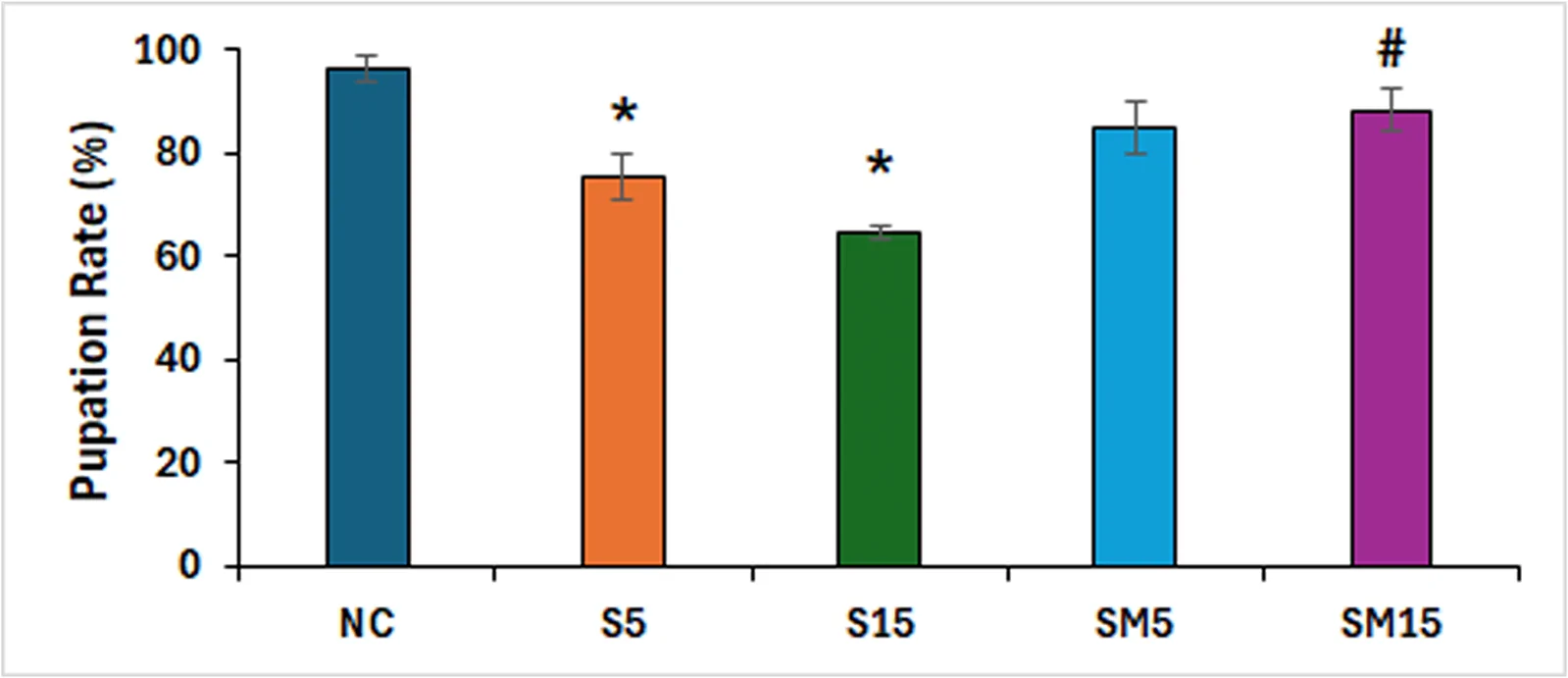
Pupation rate of *D. melanogaster.* Bar graph showing mean pupation rate (± SD) across five groups: NC (normal control), S5 (5% sucrose), S15 (15% sucrose), SM5 (5% sucrose + 5 mM metformin), and SM15 (15% sucrose + 15 mM metformin). Both sucrose-only groups showed significantly reduced pupation relative to NC. SM5 showed a non-significant increase relative to S5, whereas SM15 showed a significant improvement relative to S15. Data represent three independent biological experiments. *P < 0.05 versus NC; #P < 0.05 for S5 versus SM5 or S15 versus SM15, as applicable.

### Survival rate of *D. melanogaster*

The NC group showed the highest survival rate at the end of the 10-day treatment period, as shown in Fig 2. Flies exposed to 5% sucrose (S5) exhibited a moderate but significant reduction in survival, whereas those exposed to 15% sucrose (S15) showed a more marked significant decline. In the SM5 group, survival was modestly improved relative to S5, although the difference was not statistically significant. In contrast, the SM15 group exhibited a significant increase in survival relative to S15. These results indicate partial attenuation of sucrose-associated mortality within the corresponding treatment groups.

**Fig 2:**
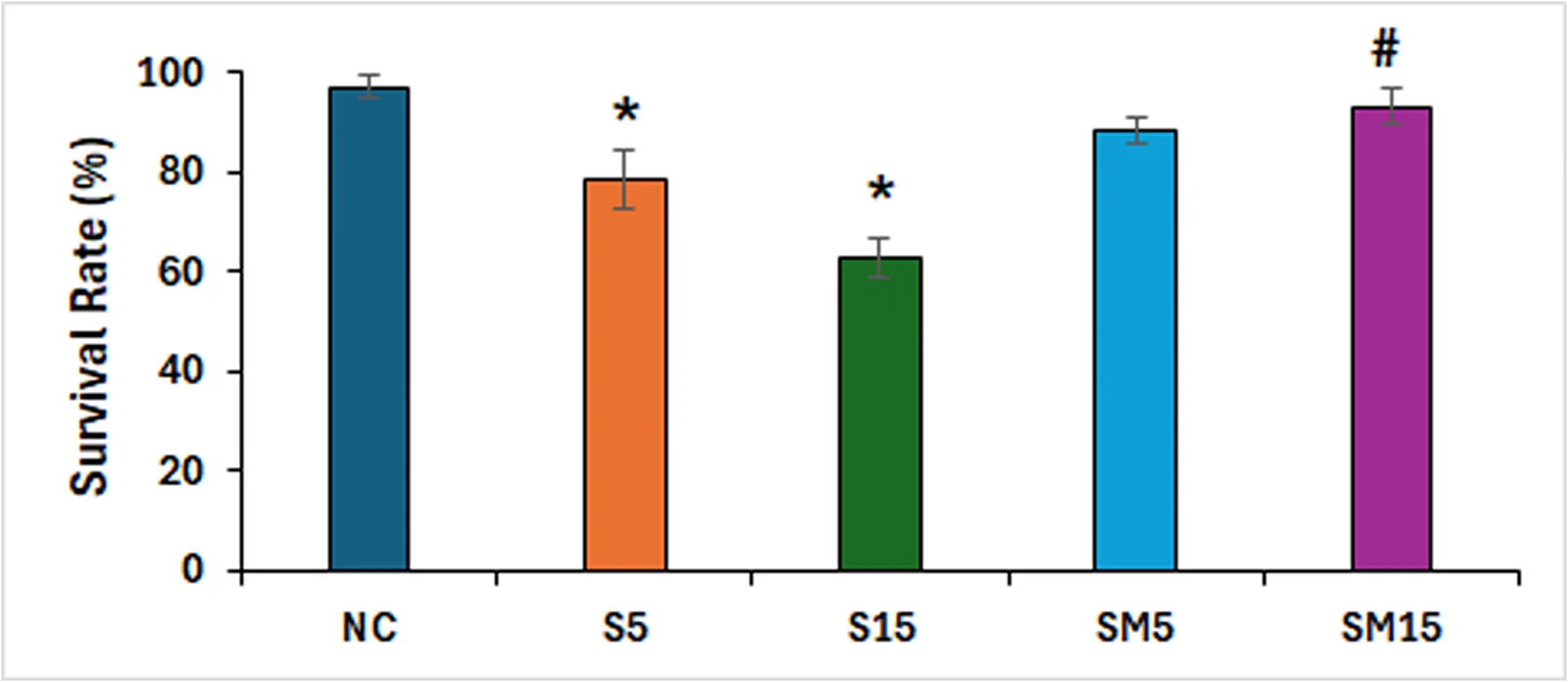
Survival rate of *D. melanogaster.* Bar graph showing mean survival rate (± SD) across NC, S5, S15, SM5, and SM15 groups. Both sucrose-only groups showed significantly reduced survival relative to NC. SM5 showed a non-significant increase relative to S5, whereas SM15 showed a significant improvement relative to S15. Data represent three independent biological experiments. *P < 0.05 versus NC; #P < 0.05 for S5 versus SM5 or S15 versus SM15, as applicable.

### Negative geotaxis and locomotor performance of *D. melanogaster*

Locomotor performance, assessed as the percentage of flies crossing the 8-cm mark within 10 s, was highest in the NC group, as shown in Fig 3. Sucrose feeding significantly impaired climbing performance in both sucrose-only groups. The SM5 group showed a modest, non-significant improvement relative to S5, whereas the SM15 group showed a significant improvement relative to S15. These results indicate that metformin partially attenuated sucrose-associated locomotor impairment within the corresponding treatment groups.

**Fig 3:**
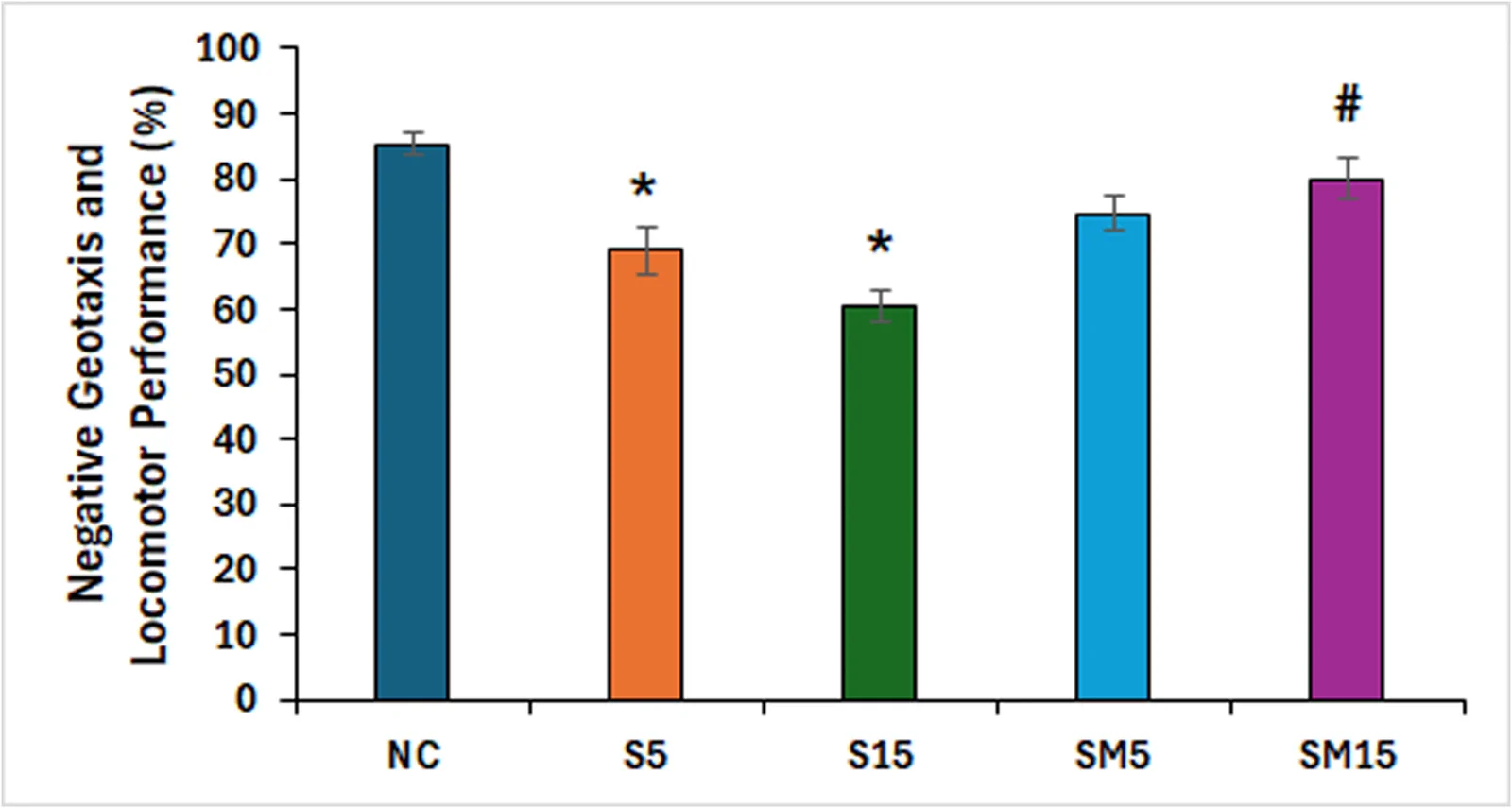
Negative geotaxis and locomotor performance in *D. melanogaster.* Bar graph showing the mean percentage of flies crossing the 8-cm mark within 10 s (± SD) across NC, S5, S15, SM5, and SM15 groups. Both sucrose-only groups showed significantly impaired climbing performance relative to NC. SM5 showed a non-significant improvement relative to S5, whereas SM15 showed a significant improvement relative to S15. Data represent three independent biological experiments. *P < 0.05 versus NC; #P < 0.05 for S5 versus SM5 or S15 versus SM15, as applicable.

### Trehalose levels of *D. melanogaster*

Trehalose levels were lowest in the NC group, as shown in Fig 4. Dietary sucrose significantly elevated trehalose concentrations in both sucrose-only groups. The SM5 group showed a modest, non-significant reduction relative to S5, whereas the SM15 group showed a significant decrease relative to S15. These findings indicate that high-sucrose diets disrupt carbohydrate homeostasis and that metformin partially attenuates trehalose accumulation within the corresponding sucrose condition.

**Fig 4:**
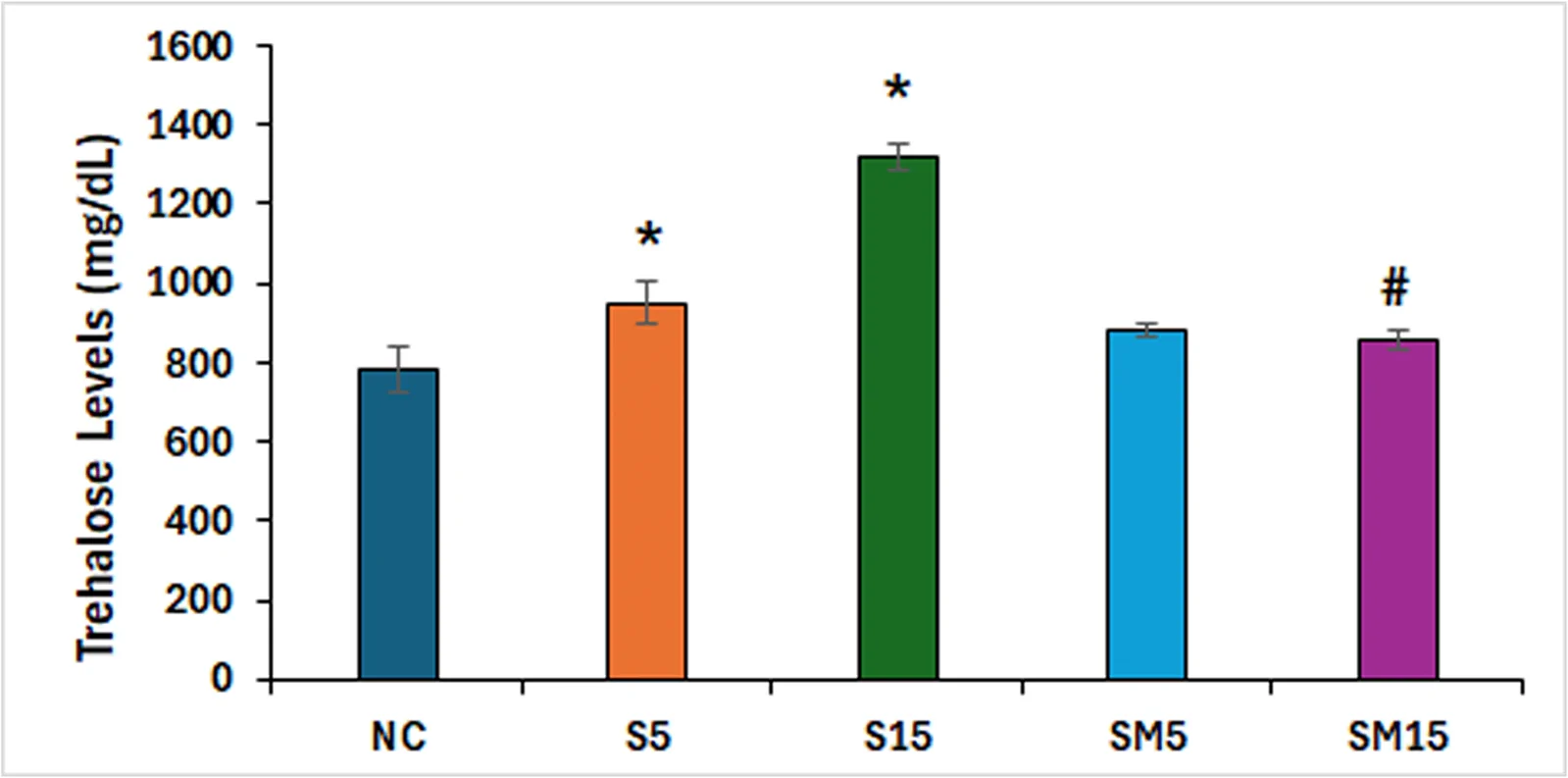
Trehalose levels in *D. melanogaster.* Bar graph displaying mean trehalose concentration (± SD) across NC, S5, S15, SM5, and SM15 groups. Both sucrose-only groups showed significantly elevated trehalose relative to NC. SM5 showed a non-significant reduction relative to S5, whereas SM15 showed a significant reduction relative to S15. Data represent three independent biological experiments. *P < 0.05 versus NC; #P < 0.05 for S5 versus SM5 or S15 versus SM15, as applicable.

### SOD activity in *D. melanogaster*

The NC group exhibited the highest SOD activity, as shown in Fig 5. Exposure to sucrose diets significantly reduced SOD activity in both sucrose-only groups. The SM5 group showed a modest, non-significant increase relative to S5, whereas the SM15 group demonstrated a significant increase relative to S15; however, activity remained below the NC level. These results indicate partial improvement of antioxidant defense within the corresponding treatment groups.

**Fig 5:**
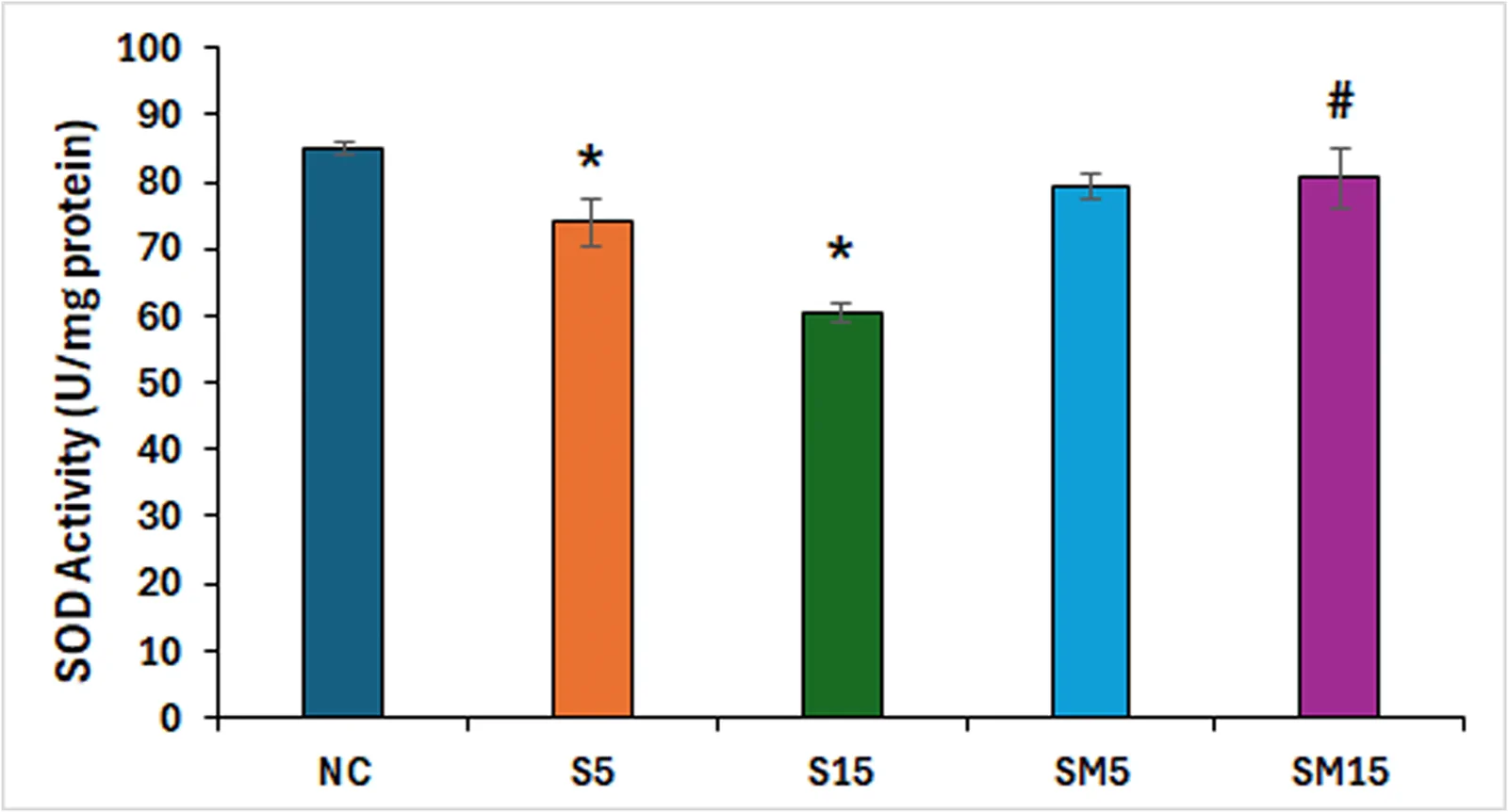
SOD activity in *D. melanogaster.* Bar graph showing mean SOD activity normalized to total protein (± SD) across NC, S5, S15, SM5, and SM15 groups. Both sucrose-only groups showed significantly reduced SOD activity relative to NC. SM5 showed a non-significant increase relative to S5, whereas SM15 showed a significant increase relative to S15, although activity remained below NC. Data represent three independent biological experiments. *P < 0.05 versus NC; #P < 0.05 for S5 versus SM5 or S15 versus SM15, as applicable.

### GST activity in *D. melanogaster*

GST activity was highest in the NC group, as shown in Fig 6. Sucrose feeding caused a significant decline in GST activity in both sucrose-only groups. The SM5 group showed a slight but non-significant improvement relative to S5, whereas the SM15 group exhibited a significant increase relative to S15. These findings indicate partial improvement of antioxidant and detoxification capacity within the corresponding treatment groups.

**Fig 6:**
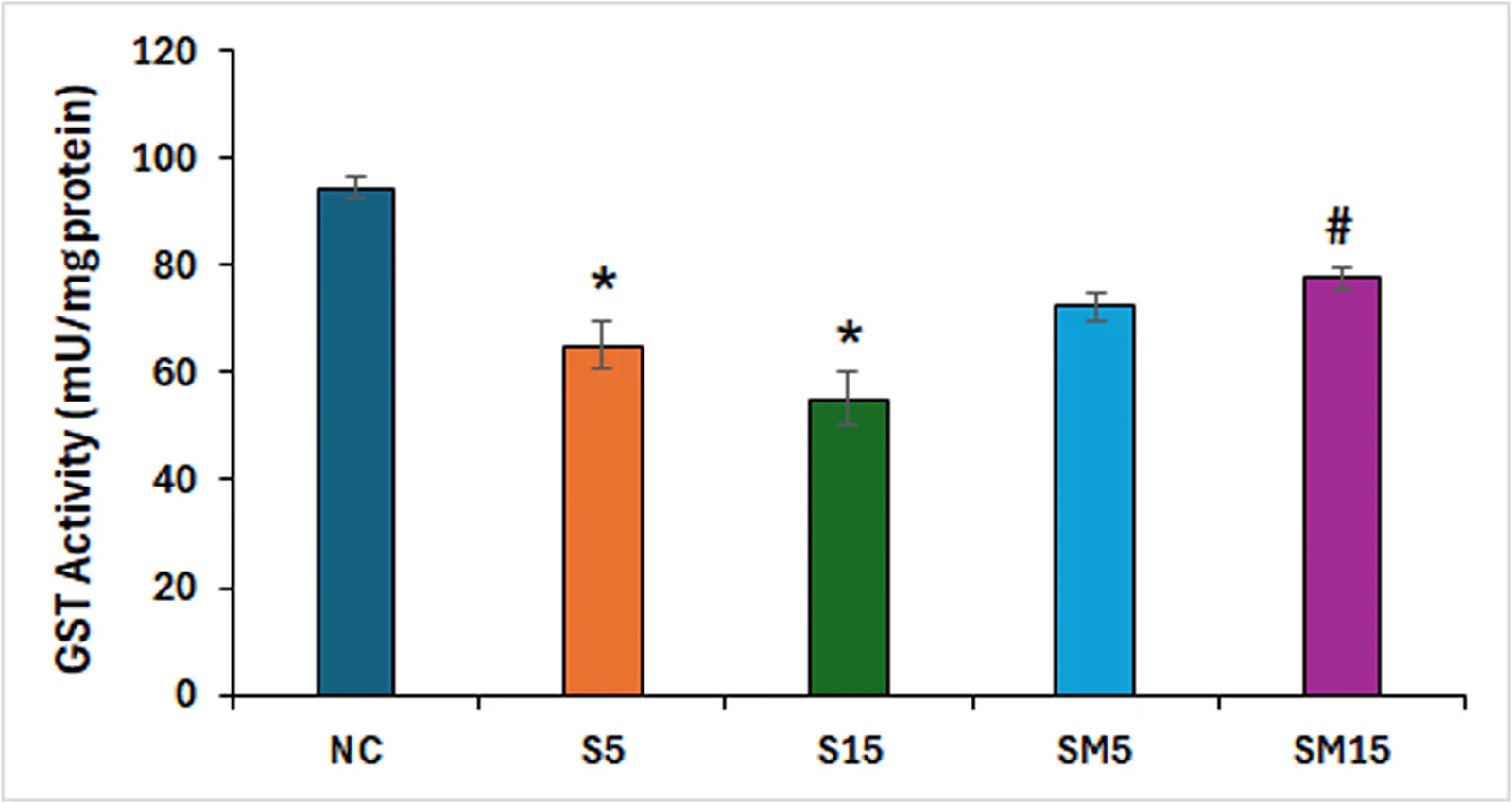
GST activity in *D. melanogaster.* Bar graph showing mean GST activity (mU/mg protein ± SD) across NC, S5, S15, SM5, and SM15 groups. Both sucrose-only groups showed significantly reduced GST activity relative to NC. SM5 showed a non-significant increase relative to S5, whereas SM15 showed a significant increase relative to S15. Data represent three independent biological experiments. *P < 0.05 versus NC; #P < 0.05 for S5 versus SM5 or S15 versus SM15, as applicable.

### Mn-SOD gene expression in *D. melanogaster*

Relative to the NC calibrator, Mn-SOD expression was significantly downregulated in both sucrose-only groups, as shown in Fig 7. The SM5 group showed a modest, non-significant increase relative to S5, whereas the SM15 group showed a significant increase relative to S15. These findings are consistent with partial improvement of mitochondrial antioxidant gene expression within the corresponding treatment groups.

**Fig 7:**
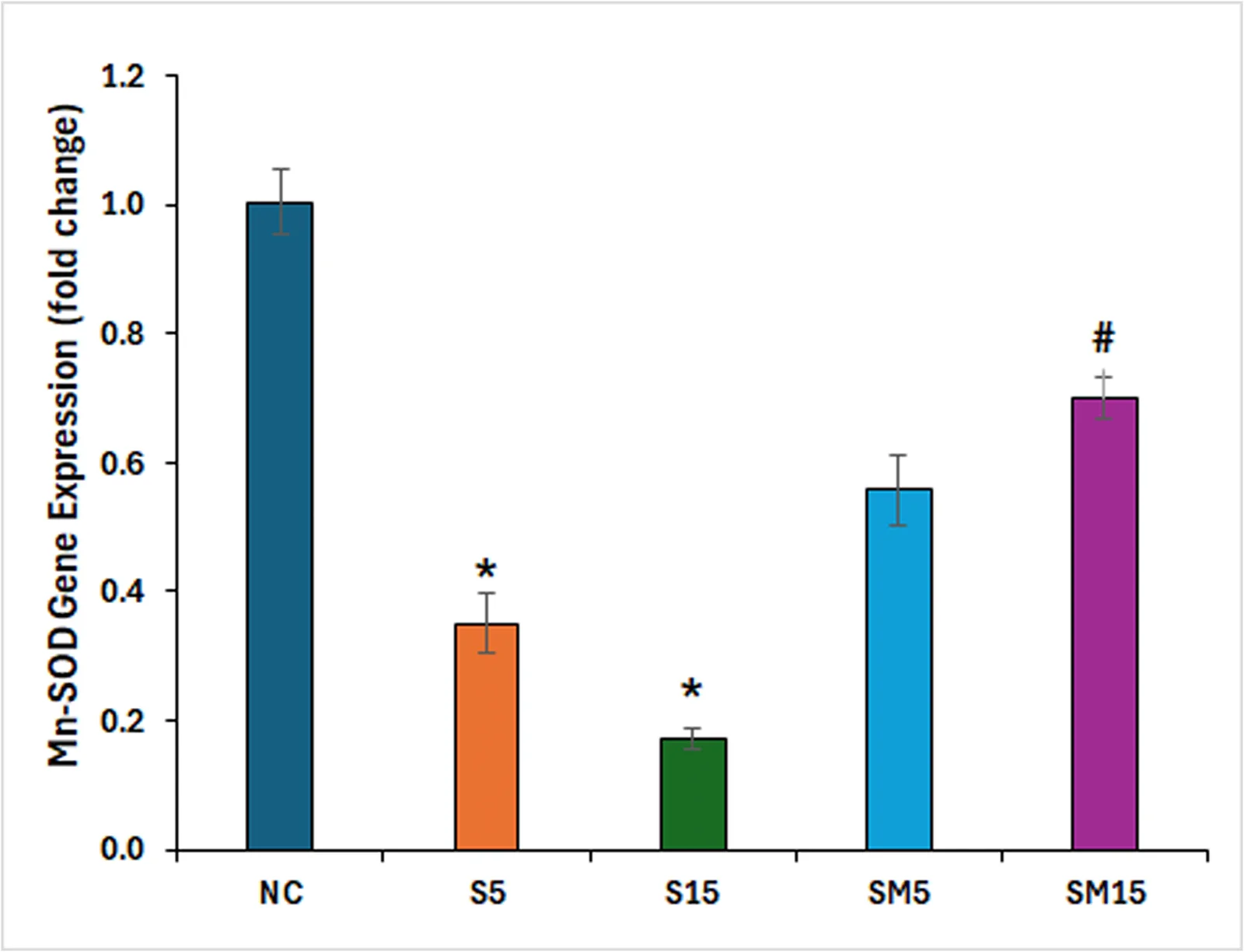
Fold change in Mn-SOD gene expression in *D. melanogaster.* Both sucrose-only groups showed significantly reduced expression relative to NC. SM5 showed a non-significant increase relative to S5, whereas SM15 showed a significant increase relative to S15. Expression was normalized to RpS20, and NC served as the calibrator. Data represent three independent biological experiments. *P < 0.05 versus NC; #P < 0.05 for S5 versus SM5 or S15 versus SM15, as applicable.

### Cu-Zn-SOD gene expression in *D. melanogaster*

Cu-Zn-SOD expression was significantly reduced in both sucrose-only groups relative to the NC calibrator, as shown in Fig 8. The SM5 group showed a non-significant increase relative to S5, whereas the SM15 group showed a significant increase relative to S15. These findings indicate partial improvement of cytosolic antioxidant gene expression within the corresponding treatment groups.

**Fig 8:**
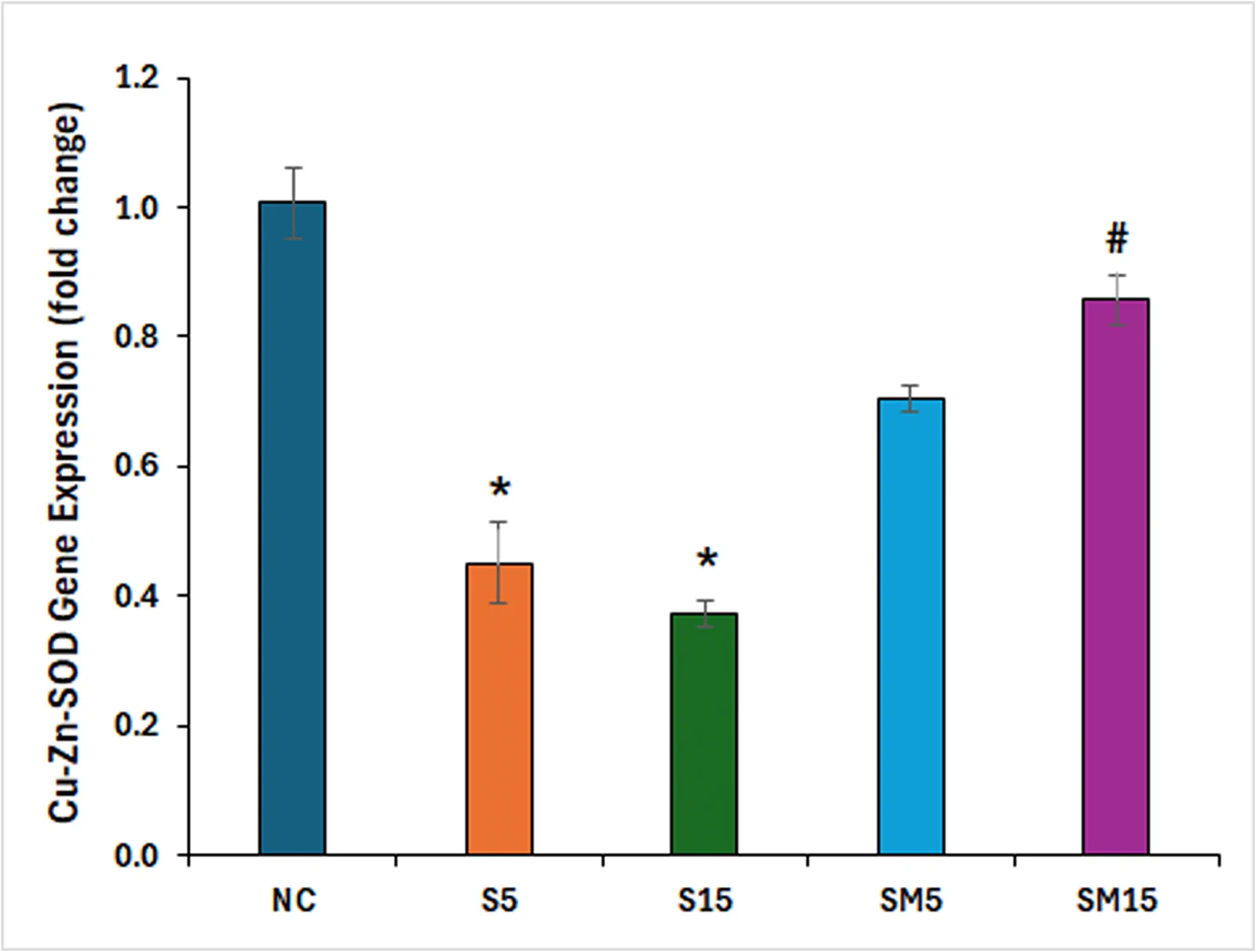
Fold change in Cu-Zn-SOD gene expression in *D. melanogaster.* Both sucrose-only groups showed significantly reduced expression relative to NC. SM5 showed a non-significant increase relative to S5, whereas SM15 showed a significant increase relative to S15. Expression was normalized to RpS20, and NC served as the calibrator. Data represent three independent biological experiments. *P < 0.05 versus NC; #P < 0.05 for S5 versus SM5 or S15 versus SM15, as applicable.

### MTH gene expression in *D. melanogaster*

MTH expression was significantly reduced in both sucrose-only groups relative to the NC calibrator, as shown in Fig 9. The SM5 group showed a partial but non-significant increase relative to S5, whereas the SM15 group showed a significant increase relative to S15. These data are consistent with partial improvement of stress-related gene expression within the corresponding treatment groups.

**Fig 9:**
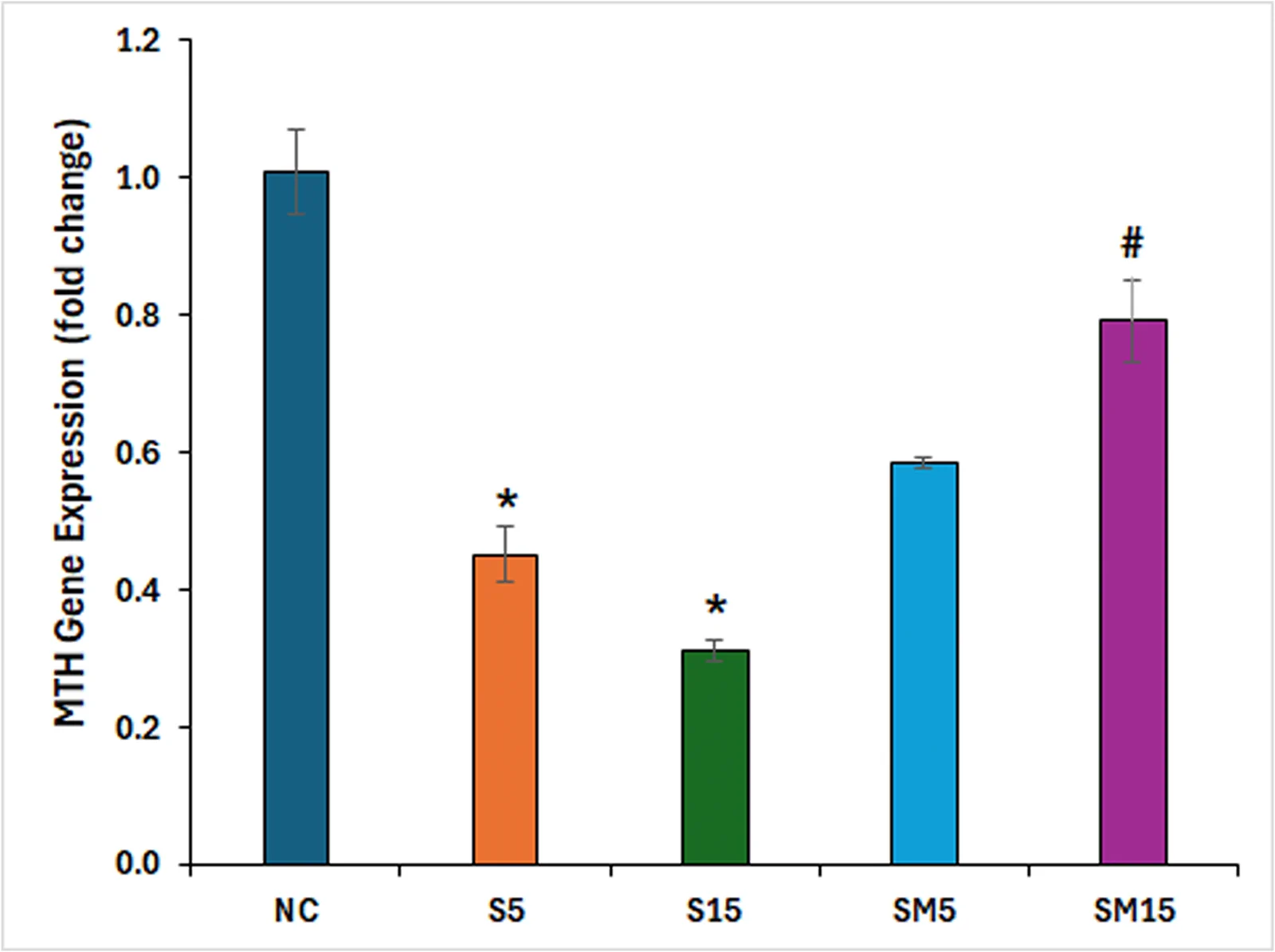
Fold change in MTH gene expression in *D. melanogaster.* Both sucrose-only groups showed significantly reduced expression relative to NC. SM5 showed a non-significant increase relative to S5, whereas SM15 showed a significant increase relative to S15. Expression was normalized to RpS20, and NC served as the calibrator. Data represent three independent biological experiments. *P < 0.05 versus NC; #P < 0.05 for S5 versus SM5 or S15 versus SM15, as applicable.

### CAT gene expression in *D. melanogaster*

CAT expression was significantly downregulated in both sucrose-only groups relative to the NC calibrator, as shown in Fig 10. The SM5 group showed a non-significant increase relative to S5, whereas the SM15 group showed a significant increase relative to S15. These findings indicate partial improvement of CAT expression within the corresponding treatment groups.

**Fig 10:**
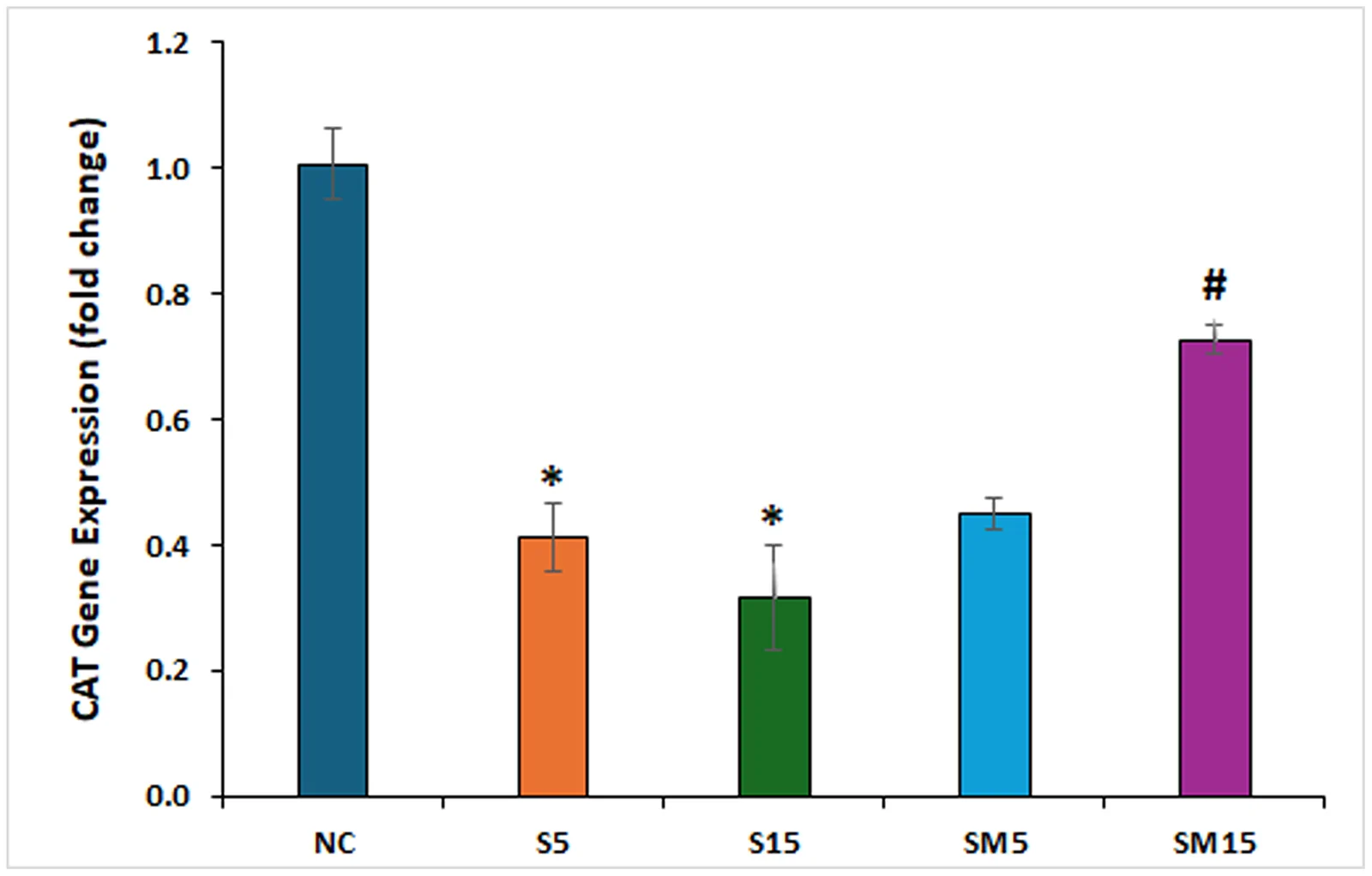
Fold change in CAT gene expression in *D. melanogaster.* Both sucrose-only groups showed significantly reduced expression relative to NC. SM5 showed a non-significant increase relative to S5, whereas SM15 showed a significant increase relative to S15. Expression was normalized to RpS20, and NC served as the calibrator. Data represent three independent biological experiments. *P < 0.05 versus NC; #P < 0.05 for S5 versus SM5 or S15 versus SM15, as applicable.

### Hsp40 gene expression in *D. melanogaster*

Hsp40 expression was significantly reduced in sucrose-fed flies compared with NC, as shown in Fig 11. The S5 group exhibited significant downregulation, while the S15 group showed a more severe decrease, indicating suppression of this molecular chaperone under dietary stress. Metformin treatment improved Hsp40 expression. The SM5 group showed a modest but non-significant increase relative to S5, whereas the SM15 group exhibited significant restoration relative to S15, while remaining different from NC. These findings indicate that metformin helps preserve stress-response capacity and proteostasis-related gene expression during sucrose challenge.

**Fig 11:**
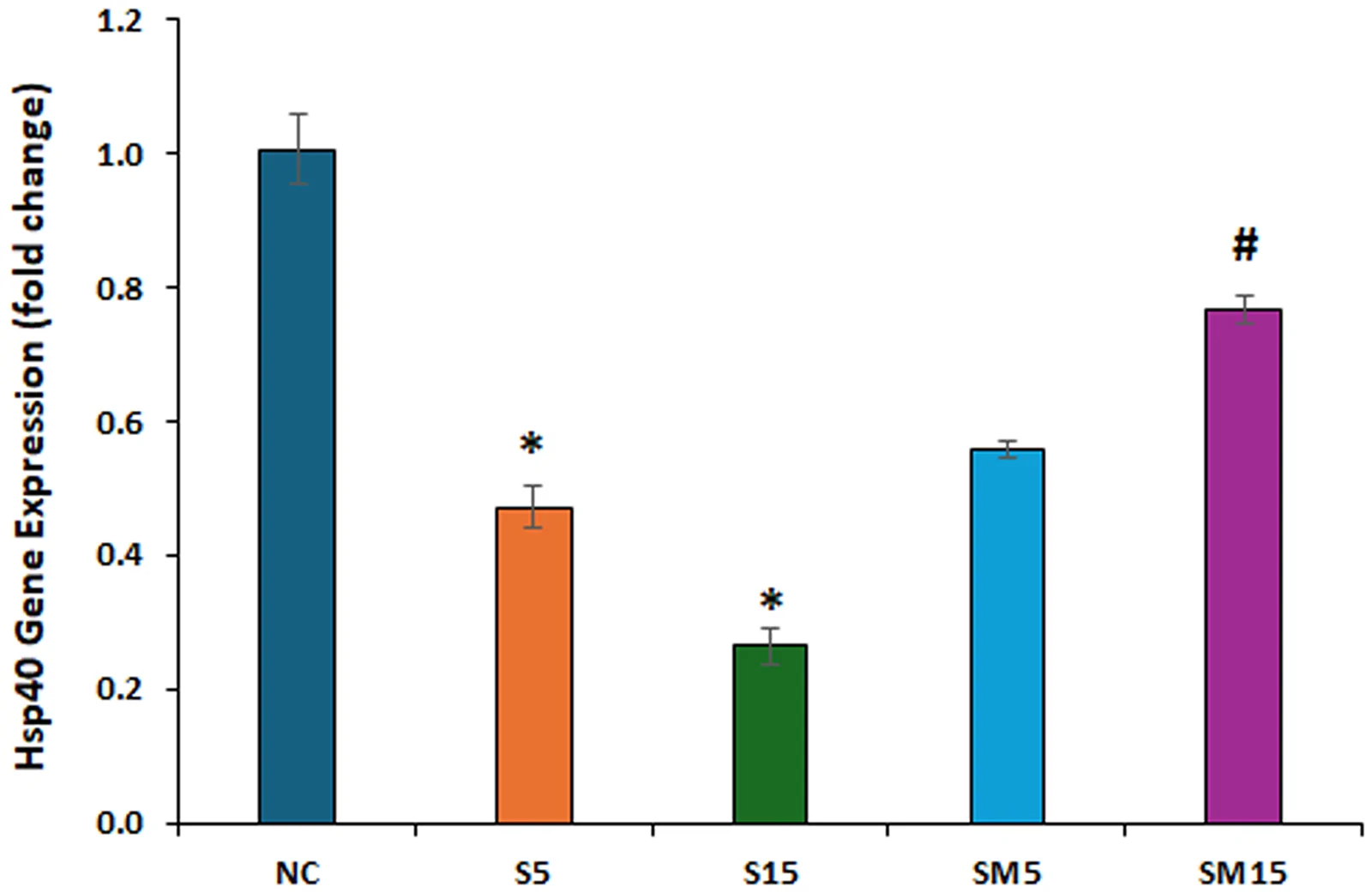
Fold change in Hsp40 gene expression in *D. melanogaster.* Both sucrose-only groups showed significantly reduced expression relative to NC. SM5 showed a non-significant increase relative to S5, whereas SM15 showed a significant increase relative to S15. Expression was normalized to RpS20, and NC served as the calibrator. Data represent three independent biological experiments. *P < 0.05 versus NC; #P < 0.05 for S5 versus SM5 or S15 versus SM15, as applicable.

### Hsp70 gene expression in *D. melanogaster*

Hsp70 expression was also significantly downregulated in sucrose-fed flies relative to the NC group, as shown in Fig 12. The decrease was significant in S5 and more pronounced in S15, suggesting progressive impairment of cellular stress tolerance with increasing sucrose exposure. Metformin treatment improved Hsp70 expression within each corresponding sucrose condition. The SM5 group showed a modest but non-significant improvement relative to S5, whereas the SM15 group showed a significant increase relative to S15. These data further support a protective role for metformin in enhancing stress tolerance under high-sucrose conditions.

**Fig 12:**
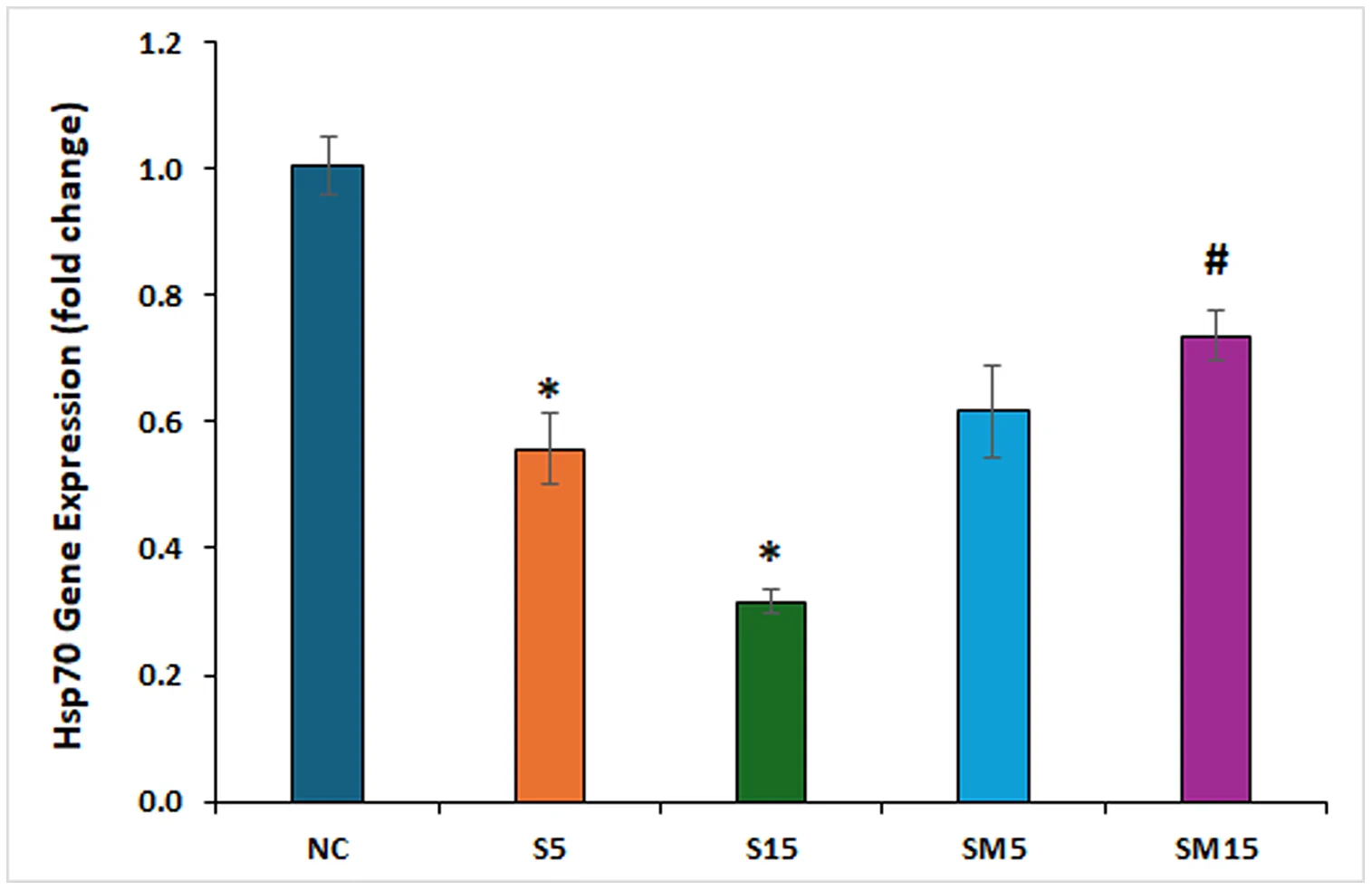
Fold change in Hsp70 gene expression in *D. melanogaster.* Both sucrose-only groups showed significantly reduced expression relative to NC. SM5 showed a non-significant increase relative to S5, whereas SM15 showed a significant increase relative to S15. Expression was normalized to RpS20, and NC served as the calibrator. Data represent three independent biological experiments. *P < 0.05 versus NC; #P < 0.05 for S5 versus SM5 or S15 versus SM15, as applicable.

## Discussion

This study provides an integrated evaluation of physiological, developmental, behavioral, biochemical, and molecular responses of *D. melanogaster* to chronic high-sucrose feeding and of the effects of metformin within each corresponding sucrose condition. High dietary sucrose was associated with reduced pupation and survival, elevated trehalose levels, impaired locomotor performance, diminished antioxidant enzyme activity, and downregulation of stress- and antioxidant-related genes. Metformin partially attenuated several of these alterations relative to the corresponding sucrose-only groups; however, most parameters did not return to NC levels. The present study was designed as a matched-intervention study rather than a full factorial sucrose × metformin experiment. Therefore, the findings should not be interpreted as evidence of a metformin dose-response relationship or a sucrose × metformin interaction.

High-sucrose diets in *Drosophila* are widely recognized for producing carbohydrate dysregulation, insulin resistance-like phenotypes, and obesity-associated traits [11,13]. In the present study, both sucrose-only diets impaired pupation and survival, indicating systemic metabolic stress and developmental disruption. The significant elevation of trehalose further supports a hyperglycemia-like state because trehalose is the principal circulating sugar in flies and reflects systemic carbohydrate imbalance [43].

These metabolic disturbances are likely associated with dysregulation of conserved insulin/insulin-like growth factor (IGF)-like signaling pathways. In D. melanogaster, insulin-like peptides (DILPs) activate the insulin receptor (InR), initiating the PI3K-AKT signaling cascade and regulating downstream effectors such as dFOXO and d4EBP, which control energy homeostasis and stress responses [43,44]. Chronic high-sucrose feeding has been reported to impair insulin pathway responsiveness, resulting in altered signaling and sustained trehalose accumulation, consistent with insulin resistance–like states in this model [9,13,17]. Because trehalose metabolism is tightly regulated by DILP-InR signaling, its elevation under high-sucrose conditions reflects systemic carbohydrate dysregulation. In parallel, excessive sugar intake promotes mitochondrial dysfunction and increased production of reactive oxygen species (ROS), contributing to oxidative stress and metabolic imbalance [45]. The role of mitochondrial ROS overproduction as a driver of hyperglycemia-induced cellular damage provides mechanistic context for the oxidative stress observed in this study [46]. The reduction in trehalose levels following metformin treatment suggests partial improvement in metabolic regulation, potentially associated with modulation of conserved metabolic pathways such as AMPK signaling [47,48], although these pathways were not directly measured.

The decline in locomotor performance observed in sucrose-fed flies indicates that metabolic dysfunction extended to functional behavior. Negative geotaxis assays showed significant reductions in climbing performance under both sucrose conditions. Previous studies have linked high-sugar feeding to neuronal and muscle oxidative damage [49,50], and lipid accumulation may impair mitochondrial efficiency [12]. In the present study, improved climbing performance in the metformin-treated groups is consistent with partial functional improvement; no structural or histological inference is made.

Oxidative stress plays a central role in the pathophysiology of metabolic disorders and contributes to cellular dysfunction through disruption of redox balance. In this study, high-sucrose feeding resulted in significant reductions in antioxidant enzyme activities, including SOD and GST, indicating compromised redox defense. SOD detoxifies superoxide radicals, while GST facilitates the removal of lipid peroxidation products and toxic metabolites [45,51,52]. The reduction in these enzymes suggests that chronic sucrose exposure disrupts cellular antioxidant capacity. This enzymatic decline was accompanied by transcriptional downregulation of antioxidant genes, particularly SOD, indicating that high-sucrose diets interfere with regulatory pathways controlling oxidative stress responses. Similar mechanisms have been described in mammalian systems, where chronic hyperglycemia activates stress-sensitive pathways such as NF-κB and MAPK, leading to suppression of antioxidant gene expression [53,54].

Metformin treatment partially attenuated high-sucrose-associated oxidative imbalance. SOD activity increased significantly in SM15 relative to S15 but remained below the NC level, while the change in SM5 relative to S5 was not significant. These findings are consistent with partial improvement of antioxidant defense. These findings are consistent with the established role of metformin as a metabolic modulator with antioxidant properties [21,55]. Although AMPK is a recognized mediator of metformin action [22,23], AMPK activity and related signaling pathways were not directly assessed in this study; therefore, these mechanisms remain inferential.

At the molecular level, metformin partially increased the expression of antioxidant and stress-response genes relative to the corresponding sucrose-only groups. Increased Mn-SOD, Cu/Zn-SOD, and CAT expression is consistent with partial improvement of redox regulation, while increased MTH, Hsp40, and Hsp70 expression is consistent with improved cellular stress responses and proteostasis. These interpretations are limited to transcriptional changes because protein abundance and pathway activation were not measured.

Mn-SOD, a mitochondrial antioxidant enzyme, was downregulated under high-sucrose conditions. Its increased expression following metformin treatment is consistent with partial improvement of mitochondrial antioxidant defense [22,55]. Similarly, Cu/Zn-SOD expression increased relative to the corresponding sucrose-only group, indicating that the observed transcriptional effects extended to cytosolic antioxidant defense [49,52].

MTH, a gene associated with stress resistance, metabolic regulation, and longevity, was downregulated in sucrose-fed flies and increased following metformin treatment, which is consistent with improved cellular stress responses [44,45,56,57]. CAT expression also increased relative to the corresponding sucrose-only group, supporting partial improvement of hydrogen peroxide detoxification capacity [22,28,51].

The molecular chaperones Hsp40 and Hsp70 were reduced under high-sucrose conditions. Their increased expression following metformin treatment is consistent with partial improvement of protein quality-control responses. These effects may involve AMPK- and mTOR-related pathways [58–60], but those pathways were not directly assessed. Increased Hsp70 expression may support cellular adaptation to metabolic stress [13,46,61,62].

Collectively, the increases in antioxidant-related genes (Mn-SOD, Cu/Zn-SOD, and CAT) and stress-response genes (MTH, Hsp40, and Hsp70) are consistent with partial improvement of redox homeostasis and proteostatic regulation. These molecular changes align with the observed improvements in survival, development, and locomotor performance [13,25,51].

Most measured parameters did not return to NC levels, indicating partial rather than complete amelioration. Direct assessment of upstream regulators, including DILP expression, insulin receptor activity, AKT phosphorylation, AMPK activation, and dFOXO localization, was beyond the scope of this study. These measurements will be required to determine whether the observed changes are mediated through conserved insulin/IGF-like or energy-sensing pathways. Future investigations targeting these pathways would provide deeper mechanistic insight into how metformin modulates metabolic regulation under chronic high-sucrose stress.

This study reinforces the value of *D. melanogaster* as a genetically tractable model for investigating diet-induced metabolic dysfunction. The integrative design incorporated survival, developmental progression, metabolic assessment, antioxidant enzyme activity, locomotor performance, and stress-related gene expression, thereby providing multi-level characterization of the observed phenotypes.

### Limitations and future directions

Several limitations should be acknowledged. First, the study used matched sucrose-metformin groups rather than a full factorial design and did not include metformin-only control groups. Consequently, the independent effect of metformin concentration, the interaction between sucrose and metformin, and the effects of metformin under the normal diet could not be evaluated. Future studies should include all sucrose × metformin combinations and metformin-only controls.

Second, the experiments were based on three independent biological experiments. Although this design provided reproducible observations, larger numbers of independent experiments would increase statistical power, particularly for modest effects such as those observed in SM5 relative to S5.

Third, mixed-sex groups were used for the negative geotaxis assay, and sex-specific or single-fly locomotor responses were not examined. Although the sex ratio and group size were held constant across treatments, future studies should test males and females separately and consider individual-fly tracking to resolve sex-specific and social-interaction effects.

Fourth, while trehalose quantification provides a well-established functional indicator of carbohydrate dysregulation in *Drosophila*, direct assessment of DILP expression, insulin receptor activation, AKT phosphorylation, AMPK activation, and dFOXO localization was not performed. Interpretations related to insulin/IGF-like signaling therefore remain indirect.

Fifth, all experimental endpoints were assessed at a single 10-day endpoint. This design captures chronic outcomes but does not resolve the temporal onset or progression of metabolic alterations or metformin-associated effects. Longitudinal measurements at earlier and intermediate time points are needed.

Sixth, structural or histological evaluation of metabolically relevant tissues was not conducted. Therefore, conclusions regarding neuromuscular or tissue-level effects are limited to functional and biochemical observations.

Seventh, body weight and adiposity were not measured; therefore, the present study cannot determine whether the hyperglycemic flies exhibited a normal-weight or elevated-weight phenotype. Future studies should measure body weight or adiposity together with glycemic and metabolic endpoints and directly compare metformin responses in hyperglycemic flies with and without an elevated-weight phenotype. Such a factorial comparison could identify shared and phenotype-specific pathways of metformin action and clarify how obesity modifies treatment responses, building on the *Drosophila* obesity model described in Ref [32]..

Additional approaches that could further strengthen this model include:

- Full factorial sucrose × metformin experiments, including metformin-only control groups
  - Tissue-specific analysis (e.g., fat body and muscle histology)
  - Apoptosis and cellular stress assays (e.g., TUNEL staining or caspase activity)
  - Expanded behavioral profiling (e.g., feeding behavior, circadian activity)
  - Lipidomic and metabolomic analyses to better characterize systemic metabolic changes

These approaches would enhance mechanistic resolution while preserving the integrative physiological framework established in the present study.

## Conclusion

High-sucrose diets in *D. melanogaster* induced metabolic disturbances characterized by reduced survival, elevated trehalose, diminished antioxidant defenses, altered stress-related gene expression, and impaired locomotor performance.

Metformin treatment partially attenuated several high-sucrose-associated alterations relative to the corresponding sucrose-only groups, including changes in survival, metabolic parameters, antioxidant enzyme activity, locomotor performance, and stress-related gene expression. Because the design was not factorial, these findings do not establish a metformin dose-response relationship or a sucrose × metformin interaction.

The observed changes in antioxidant enzyme activity and gene expression are consistent with partial improvement of cellular stress responses, although the underlying molecular pathways were not directly investigated.

Overall, this study supports the utility of *D. melanogaster* as an accessible model for investigating diet-induced metabolic alterations and pharmacological modulation while emphasizing the need for factorial designs, larger independent replication, sex-specific behavioral analysis, longitudinal assessment, tissue-level validation, and direct interrogation of insulin/IGF-like signaling pathways.

## Supporting information

S1 Data Physiological and biochemical data. (PDF)

S2 Data Mn-SOD qRT-PCR data. (XLSX)

S3 Data Cu/Zn-SOD qRT-PCR data. (XLSX)

S4 Data MTH qRT-PCR data. (XLSX)

S5 Data CAT qRT-PCR data. (XLSX)

S6 Data Hsp40 qRT-PCR data. (XLSX)

S7 Data Hsp70 qRT-PCR data. (XLSX)

S8 Data RpS20 Reference-Gene qRT-PCR data. (XLSX)

S1 File Graphical abstract. (TIF)
