# Supplementary material for: Metformin modulates survival, oxidative stress, and gene expression in a high-sucrose Drosophila diabetes model
Source: PLoS One. 2026 Oct 1;21(10):e0359638. doi: 10.1371/journal.pone.0359638 (PMC13630249; doi:10.1371/journal.pone.0359638)
Supplement: Supplementary file 1 [file pone.0359638.s001.pdf]

1. Pupation Rate (%)

Group	Replicate 1	Replicate 2	Replicate 3	Average	SD	p-value
NC	99.000	96.000	94.000	96.333	2.517	1.000
S5	71.000	75.000	80.000	75.333	4.509	0.002
S15	66.000	63.000	65.000	64.667	1.528	0.000
SM5	80.000	90.000	85.000	85.000	5.000	0.068
SM15	87.000	93.000	85.000	88.333	4.163	0.001

2. Survival Rate (%)

Group	Replicate 1	Replicate 2	Replicate 3	Average	SD	p-value
NC	95.000	97.000	99.000	97.000	2.000	1.000
S5	81.000	72.000	83.000	78.667	5.859	0.007
S15	60.000	67.000	61.000	62.667	3.786	0.000
SM5	85.000	89.000	90.000	88.000	2.646	0.066
SM15	94.000	89.000	96.000	93.000	3.606	0.001

3. Negative Geotaxis and Locomotor Performance (%)

Group	Replicate 1	Replicate 2	Replicate 3	Average	SD	p-value
NC	85.000	87.000	84.000	85.333	1.528	1.000
S5	68.000	66.000	73.000	69.000	3.606	0.002
S15	60.000	63.000	58.000	60.333	2.517	0.000
SM5	72.000	75.000	77.000	74.667	2.517	0.089
SM15	80.000	77.000	83.000	80.000	3.000	0.001

4. Trehalose Levels (mg/dL)

Group	Replicate 1	Replicate 2	Replicate 3	Average	SD	p-value
NC	750.000	752.000	848.000	783.333	56.012	1.000
S5	965.000	992.000	889.000	948.667	53.407	0.021
S15	1351.000	1289.000	1322.000	1320.667	31.021	0.000
SM5	859.000	888.000	890.000	879.000	17.349	0.098
SM15	888.000	845.000	843.000	858.667	25.423	0.000

5. SOD Activity (U/mg protein)

Group	Replicate 1	Replicate 2	Replicate 3	Average	SD	p-value
NC	86.000	84.000	85.000	85.000	1.000	1.000
S5	77.000	75.000	70.000	74.000	3.606	0.007
S15	60.000	62.000	59.000	60.333	1.528	0.000
SM5	81.000	77.000	80.000	79.333	2.082	0.091
SM15	76.000	81.000	85.000	80.667	4.509	0.002

6. GST Activity (mU/mg)

Group	Replicate 1	Replicate 2	Replicate 3	Average	SD	p-value
NC	96.000	92.000	95.000	94.333	2.082	1.000
S5	63.000	70.000	62.000	65.000	4.359	0.000
S15	50.000	60.000	55.000	55.000	5.000	0.000
SM5	75.000	70.000	72.000	72.333	2.517	0.065
SM15	76.000	80.000	77.000	77.667	2.082	0.002
